# Stem cell therapy targets THBS1 to reverse endometrial fibrosis

**DOI:** 10.3389/fcell.2026.1766915

**Published:** 2026-02-05

**Authors:** Xiaochuan Yu, Lijuan Shi, MingBo Qu, Yonggang Zhang, Yating Zhang, Xinyu Xu, Lina Liu, Huanan Wang, Huali Wang

**Affiliations:** 1 Dalian Medical University, Dalian, China; 2 Dalian Women and Children’s Medical Center (Group), Dalian, China; 3 Dalian University of Technology, Dalian, China

**Keywords:** fibrosis, intrauterine adhesions, mesenchymal stem cells, PI3K/Akt signaling pathway, thrombospondin-1

## Abstract

**Background:**

Intrauterine adhesions (IUA) are a major cause of female infertility and recurrent pregnancy loss, characterized by endometrial fibrosis. The molecular mechanisms underlying IUA fibrosis are poorly understood. Thrombospondin-1 (THBS1), a matricellular protein linked to fibrotic disorders, has not been extensively studied in IUA. This study investigates the role of THBS1 in IUA pathogenesis and the therapeutic potential of decidua-derived mesenchymal stem cells (DMSCs), focusing on the PI3K/AKT signaling pathway.

**Methods:**

Transcriptomic profiling identified significant upregulation of THBS1 in IUA tissues. Pathway analysis suggested that THBS1 promotes fibrosis via the PI3K/AKT pathway. *In vitro* and *in vivo* IUA models were used to evaluate fibrosis markers and signaling molecules using qPCR and Western blotting. The therapeutic effects of DMSCs and THBS1 knockdown were assessed through histological analyses (H&E and Masson staining) and quantification of inflammation and angiogenesis markers.

**Results:**

THBS1 silencing reduced fibrotic markers and inhibited PI3K/AKT pathway activation *in vitro*. DMSC treatment showed a more pronounced anti-fibrotic effect, suggesting that DMSC-mediated repair involves THBS1 regulation. *In vivo*, both THBS1 knockdown and DMSC administration alleviated intrauterine fibrosis, reduced inflammation, and enhanced angiogenesis. Histological evaluations confirmed improved endometrial structure and reduced collagen deposition, especially in DMSC-treated and combination therapy groups.

**Conclusion:**

THBS1 is a key pro-fibrotic factor in IUA, modulating the PI3K/AKT pathway. DMSCs effectively mitigate fibrosis and promote endometrial regeneration, potentially by downregulating THBS1. This study highlights THBS1 as a promising therapeutic target and reinforces the clinical potential of DMSCs for IUA treatment.

## 1 Introduction

### 1.1 Background and clinical challenges of intrauterine adhesions (IUA)

Intrauterine adhesions (IUA), commonly referred to as Asherman’s syndrome, are characterized by pathological alterations resulting from mechanical trauma or infection-induced endometrial regeneration disorders, leading to the formation of fibrous tissue within the uterine cavity. This pathological condition disrupts the normal architecture and function of the endometrium, leading to various reproductive disorders, including menstrual irregularities, infertility, and recurrent pregnancy loss (Sun et al., 2024; Lee et al., 2021; Wu et al., 2023; Liu et al., 2023). Currently, the standard treatment for IUA involves hysteroscopic adhesiolysis combined with intrauterine device (IUD) or balloon placement, followed by postoperative estrogen therapy (Rodríguez-Eguren et al., 2024). However, postoperative recurrence rates remain significantly high, ranging from 20% to 62.5%, while subsequent pregnancy rates are as low as 33.3% (Ma et al., 2021; Yi et al., 2023). These challenges pose a substantial threat to female reproductive health and quality of life. Therefore, a deeper understanding of the regulatory mechanisms underlying endometrial fibrosis is urgently needed to develop more effective and durable therapeutic strategies.

### 1.2 THBS1: a key regulatory factor in endometrial fibrosis

**FIGURE 1:**
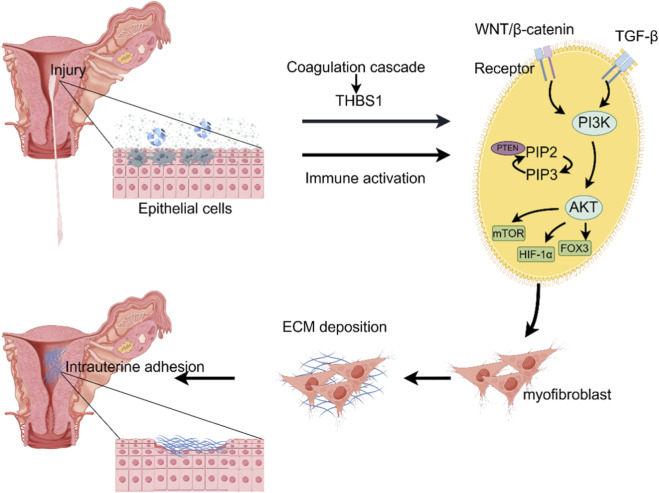
The role of the THBS1 gene in regulating the PI3K/AKT pathway in intrauterine adhesions.

Endometrial fibrosis is a defining pathological feature of intrauterine adhesions (IUA), characterized by aberrant fibroblast activation, excessive extracellular matrix (ECM) deposition, and impaired tissue remodeling. Increasing evidence identifies thrombospondin-1 (THBS1), a multifunctional matricellular glycoprotein, as a potent driver of fibrogenesis by promoting fibroblast differentiation and ECM protein synthesis (Wei et al., 2024; Murphy-Ullrich and Suto, 2018; Han et al., 2024; Yao et al., 2024). Its pro-fibrotic role has been established in the liver, lung, heart, and kidney (Wei et al., 2024; Zhou et al., 2023; Allaire et al., 2023; Yang et al., 2024). However, the specific contribution and regulatory mechanisms of THBS1 in endometrial fibrosis remain largely unknown and require systematic investigation (Figure 1).

### 1.3 Central role of the PI3K/AKT signaling pathway in tissue fibrosis

The phosphoinositide 3-kinase/protein kinase B (PI3K/AKT) signaling pathway represents a crucial intracellular cascade activated by various growth factors and cytokines. It regulates essential cellular functions, including proliferation, survival, metabolism, and anti-apoptotic responses (Deng et al., 2024). Extensive studies have demonstrated that the activation of the PI3K/AKT pathway significantly contributes to the development of fibrosis across multiple organs, such as the lungs, liver, and kidneys, by promoting fibroblast proliferation, migration, and the synthesis and deposition of collagen (Qin et al., 2021; Huang et al., 2024; Hu et al., 2020; Huang et al., 2023; Wang et al., 2024). Furthermore, PI3K/AKT signaling is known to interact synergistically with the TGF-β1 pathway, thereby amplifying fibrotic responses (Yao et al., 2024). Despite its well-established role in systemic fibrosis, the specific involvement of the PI3K/AKT pathway in endometrial fibrosis—especially in the context of THBS1 regulation—remains poorly understood and lacks direct experimental validation (Figure 1).

### 1.4 DMSCs in endometrial regeneration: potential and unresolved mechanisms

In recent years, mesenchymal stem cells (MSCs) have attracted considerable attention for their anti-inflammatory, anti-fibrotic, and immunomodulatory properties, positioning them as promising candidates for tissue regeneration and fibrosis therapy (Cen et al., 2022; Lin et al., 2023; Liu H. et al., 2024; Hua et al., 2022; Yu et al., 2024). Preclinical studies using bone marrow-, adipose-, and umbilical cord–derived MSCs have shown beneficial effects in endometrial repair (Lin et al., 2023; Cao et al., 2025). However, their clinical application is limited by restricted donor sources or invasive harvesting procedures. Our previous work demonstrated that decidua-derived MSCs (DMSCs) achieve superior outcomes in endometrial regeneration (Dutta Gupta et al., 2023; Abumaree et al., 2019; Yu et al., 2025). DMSCs secrete a cytokine profile highly conducive to immune regulation, inflammation suppression, and tissue repair (Zheng et al., 2020; Ning et al., 2024; Chen et al., 2022; Fu et al., 2017; Almutairi et al., 2024), and exhibit enhanced survival and functional activity within the uterine microenvironment, which has been linked to favorable pregnancy outcomes (Chen et al., 2022). Nevertheless, the precise anti-fibrotic targets of MSCs remain unclear. Whether combining MSC therapy with targeted molecular interventions, such as THBS1 silencing, could provide a more effective strategy for intrauterine adhesion treatment has not yet been reported (Yao et al., 2019).

### 1.5 Research aims and significance

In this study, high-throughput transcriptomic sequencing revealed, for the first time, that THBS1 is significantly upregulated in the endometrial tissues of patients with intrauterine adhesions (IUA) and is closely associated with the activation of the PI3K/AKT signaling pathway. Building on this finding, we established both *in vitro* and *in vivo* models of IUA to systematically investigate the mechanistic role of THBS1 in promoting endometrial fibrosis via the PI3K/AKT signaling axis. Furthermore, we evaluated the therapeutic potential and synergistic effects of combining THBS1 inhibition with mesenchymal stem cell (MSC) therapy.

By elucidating the contribution of the THBS1–PI3K/AKT pathway to the progression of IUA, this study aims to identify novel molecular targets and integrated intervention strategies for the precise treatment of IUA. Our findings are anticipated to provide a robust theoretical foundation and experimental evidence for the clinical translation of targeted and stem cell-based therapies in reproductive medicine.

## 2 Materials and methods

This study has been reported in accordance with the ARRIVE guidelines 2.0 to ensure transparency and reproducibility in the design, implementation, and reporting of the research.

### 2.1 Transcriptome analysis

#### 2.1.1 Sample collection and transcriptome sequencing

Endometrial tissue samples were collected from patients diagnosed with intrauterine adhesions (n = 5) and from age-matched control patients (n = 5). Transcriptome sequencing was conducted to profile the global gene expression patterns. The use of all endometrial tissue samples and control samples was obtained with written informed consent from the patients or their legal guardians/authorized representatives,was approved by the Medical Ethics Committee of Dalian Women and Children’s Medical Center (Group). The approval number: FEJT-KY-2025-12.

#### 2.1.2 GEO database retrieval

The GEO datasets (https://www.ncbi.nlm.nih.gov/geo/) were queried using the terms ‘Asherman syndrome’, ‘intrauterine adhesion’, and ‘endometrial fibrosis’. Subsequently, the datasets GSE224093 and GSE165321 were selected for further bioinformatics analysis.

#### 2.1.3 Bioinformatics data analysis

The limma package was utilized to conduct differential expression analysis on the normalized expression matrices. IUA-associated differentially expressed genes (DEGs) were defined as genes exhibiting |log2 (fold change)| > 1, with a significance threshold set at p 0.7). The network was then imported into Cytoscape, where hub genes were ranked using the CytoHubba plugin based on the Maximal Clique Centrality (MCC) algorithm.

### 2.2 Cell culture and establishment of the IUA cell model

#### 2.2.1 Cell culture

An immortalized human endometrial stromal cell line (IHESC; ZQY064, Zhongqiaoxinzhou) was cultured in DMEM/F12 medium (G4612, Servicebio), supplemented with 10% fetal bovine serum (FBS) and 1% penicillin-streptomycin. The cells were maintained at 37 °C in a humidified atmosphere containing 5% CO_2_. The culture medium was refreshed every 3 days, and the cells were passaged using 0.25% trypsin (KGY0012, KeyGEN BioTECH).

Primary human decidual mesenchymal stromal cells (hDMSCs) were sourced from the Shenyang Cell Engineering Technology Research & Development Center Co. Ltd. The cells were cultured in DMEM medium (G4515, Servicebio) supplemented with 10% fetal bovine serum (FBS) and 1% penicillin-streptomycin under standard conditions of 37 °C and 5% CO_2_. The culture medium was replaced every 3 days, and cells were passaged using 0.25% trypsin.

#### 2.2.2 Induction of the IUA cell model

Human endometrial stromal cells (hESCs) were seeded and cultured until they reached approximately 80% confluence. Subsequently, the cells were treated with various concentrations of TGF-β1 (0, 20, 40, and 60 ng/mL; HY-P7118, MCE) for a duration of 72 h. The expression levels of fibrosis-associated markers were assessed using quantitative real-time PCR (qRT–PCR) and Western blot (WB). The concentration that induced the highest degree of fibrosis was selected to establish an *in vitro* model of intrauterine adhesion (IUA) for subsequent experiments.

#### 2.2.3 siRNA-mediated knockdown of THBS1

Small interfering RNAs (siRNAs) targeting THBS1 were synthesized by GenePharma (Shanghai, China). Cells were seeded at a density of 1 × 10^5^ cells per well in 6-well plates and cultured at 37 °C with 5% CO_2_ until they reached 70%–90% confluence. Transfection was performed according to the manufacturer’s instructions, using 5 μL of transfection reagent (40802ES03, Yeasen) and 100 pmol of siRNA per well. Six hours post-transfection, the culture medium was replaced with fresh growth medium. Total RNA was extracted after 24 h for quantitative reverse transcription polymerase chain reaction (qRT–PCR), and total protein was harvested after 48 h for Western blot analysis.

The siRNA sequences are listed in Table 1.

**TABLE 1:** The siRNA sequences.

Target	Sequence (5′-3′)
THBS1 (siRNA1) sense	GCA​UGA​CCC​UCG​UCA​CAU​ATT
THBS1 (siRNA1) antisense	UAU​GUG​ACG​AGG​GUC​AUG​CTT
THBS1 (siRNA2) sense	CCA​ACA​AAC​AGG​UGU​GCA​ATT
THBS1 (siRNA2) antisense	UUG​CAC​ACC​UGU​UUG​UUG​GTT
THBS1 (siRNA3) sense	GGU​UUC​CUC​CUU​CUG​GCA​UTT
THBS1 (siRNA3) antisense	AUG​CCA​GAA​GGA​GGA​AAC​CTT
THBS1 (siRNA4) sense	GCG​UGA​AGU​GUA​CUA​GCU​ATT
THBS1 (siRNA4) antisense	UAG​CUA​GUA​CAC​UUC​ACG​CTT
NC sense	UUC​UCC​GAA​CGU​GUC​ACG​UTT
NC antisense	ACG​UGA​CAC​GUU​CGG​AGA​ATT

### 2.3 Effects of THBS1 and DMSC treatment on the in vitro IUA model

Cells were divided into the following experimental groups: (1) control group (untreated normal cells), (2) IUA group (cells subjected to *in vitro* IUA modeling), (3) THBS1 knockdown group (IUA model cells transfected with *THBS1* siRNA), (4) DMSC group (IUA model cells treated with decidual mesenchymal stromal cells), and (5) DMSC + THBS1 knockdown group (IUA model cells treated with both *THBS1* siRNA and DMSCs).

Quantitative real-time PCR (qRT–PCR) and Western blot (WB) analyses were conducted to assess THBS1 expression, fibrosis-related markers, and the activation of the PI3K/AKT signaling pathway across the various groups.

### 2.4 Establishment and treatment of the IUA animal model

#### 2.4.1 Establishment of the IUA rat model

All animal procedures were approved by the Ethics Committee of Dalian University of Technology (Approval No. DUTSBE250305-01). Female Sprague-Dawley rats (8 weeks old, 200–220 g) were procured from the Animal Experimental Center of Dalian Medical University. The rats were housed at 22 °C under a 12-h light/12-h dark cycle. At study termination, all animals were euthanized under deep anaesthesia by individual exposure to 5% isoflurane in O_2_ (flow rate 1 L min^-1^) within a transparent induction chamber for 3–5 min until loss of the righting reflex was confirmed.

A total of nine rats were randomly divided into three groups. An intrauterine adhesion (IUA) model was established through a combination of mechanical and chemical injury. Briefly, the rats were anesthetized using 4%–5% isoflurane for induction and maintained under 1%–2% isoflurane. Following disinfection, a midline laparotomy was performed to expose the uterus. The endometrium was gently scraped using a small curette for approximately ten cycles, followed by intrauterine irrigation with 75% ethanol to induce endometrial damage. The uterine cavity was subsequently flushed with phosphate-buffered saline (PBS), and the uterus and abdominal wall were sutured.

On postoperative days 3, 7, and 10 (n = 3 per time point), uterine tissues were collected for analysis. Fibrosis-related gene expression was assessed using reverse transcription quantitative polymerase chain reaction (RT-qPCR), while protein expression was analyzed through Western blotting. The time point that exhibited the most pronounced fibrosis was selected for subsequent experiments.

#### 2.4.2 Establishment of THBS1 knockdown in rats

Nine additional 8-week-old female Sprague-Dawley rats were randomly assigned to three groups (n = 3 per group): control, AAV (THBS1 knockdown), and negative control (NC). The rats in the AAV group received an intrauterine injection of 4.1 × 10^11^ viral particles encoding siRNA targeting THBS1, while the NC group received an equivalent dose of an empty vector. Two weeks post-injection, uterine tissues were collected for quantitative reverse transcription PCR (qRT–PCR) and Western blot analyses to confirm the efficiency of THBS1 knockdown at both the mRNA and protein levels.

#### 2.4.3 Effects of THBS1 knockdown and DMSC treatment on the IUA rat model

Thirty 8-week-old female Sprague-Dawley rats were randomly assigned to five groups (n = 6 per group): (1) control group (untreated healthy rats), (2) IUA model group, (3) IUA + AAV group (intrauterine injection of AAV-THBS1), (4) IUA + DMSC group (intrauterine injection of DMSCs), and (5) IUA + DMSC + THBS1 knockdown group (sequential intrauterine injection of AAV-THBS1 followed by DMSCs).

At 7 and 14 days post-treatment, the rats were euthanized, and uterine tissues were harvested. Histological evaluations were performed using hematoxylin and eosin (H&E) staining and Masson’s trichrome staining. The extent of fibrosis resolution was further assessed through RT–qPCR and Western blot analysis of fibrosis-related markers.

### 2.5 Histological evaluation

Uterine tissues were harvested from experimental rats and subsequently fixed in 4% paraformaldehyde. Following paraffin embedding, tissue sections were prepared for histological analysis. Hematoxylin and eosin (H&E) staining, as well as Masson’s trichrome staining, were conducted to evaluate endometrial regeneration and the degree of fibrosis, respectively. H&E staining was utilized to assess endometrial architecture and to quantify the number of endometrial glands, while Masson’s trichrome staining was employed to differentiate collagen fibers. The extent of fibrosis was determined by calculating the ratio of collagen fiber area to the total tissue area.

### 2.6 Molecular analyses

#### 2.6.1 qRT–PCR

Total RNA was extracted from uterine tissues using the RNAeasy™ Animal RNA Extraction Kit (R0027, Beyotime) in accordance with the manufacturer’s protocol. Complementary DNA (cDNA) was synthesized from the extracted total RNA utilizing a reverse transcription kit (11141ES60, Yeasen). Gene expression levels were quantified employing SYBR Green-based quantitative PCR (qPCR) (11184ES25, Yeasen). The expression levels of THBS1, along with fibrosis-related and inflammation-associated genes, were evaluated. β-Actin served as the internal reference gene, and relative expression levels were calculated using the 2^−^ΔΔCt method.

Primer sequences used for qRT–PCR are listed in Table 2.

**TABLE 2:** Primer sequences used for qRT–PCR.

Gene	Species	Forward primer (5′–3′)	Reverse primer (5′–3′)
THBS1	Human	TTT​GGC​TAC​CAG​TCC​AGC​AG	AGA​AAG​GCC​CGA​GTA​TCC​CT
Collagen	Human	AGGGCTGGGCGGGAGAG	GAC​ACA​TCA​AGA​CAA​GAA​CGA​GGT​AG
Fibronectin	Human	ATA​ATC​ATA​GCC​TCA​CAG​CAG​TAA​CAG	CTT​CTC​GTG​GTG​CCT​CTC​CTC
SMA	Human	AAG​GAA​AGG​CAG​AGA​TAA​TGG​CTA​AC	GAG​ACA​AGA​AGT​GGA​ATG​GAG​AAG​G
THBS1	Rat	TTG​TCT​TTG​GAA​CCA​CAC​CA	CTG​GAC​AGC​TCA​TCA​CAG​G
Actin	Human	TGG​GCA​TGG​GTC​AGA​AGG​ATT​C	GCA​GCT​CAT​TGT​AGA​AGG​TGT​GG
Actin	Rat	GGT​GTG​ATG​GTG​GGT​ATG​GG	CAA​TGC​CGT​GTT​CAA​TGG​GG
Collagen	Rat	TAG​GAG​TCG​AGG​GAC​CCA​AG	GTT​TCC​TCC​AAG​ACC​AGG​GG
Fibronectin	Rat	CGA​TGC​CAT​TCC​AGC​CAA​TG	GTT​GAG​CGT​GTA​CAG​GTG​GA
SMA	Rat	CGG​AAG​TCA​AGA​GGC​TGT​GT	CGT​CTG​GAC​AGT​CTG​CAG​TT
VEGF	Rat	CTC​CGT​AGT​AGC​CGT​GGT​CTG	CTT​CTC​TTC​CTC​CCC​TCT​CTT​CTC
αβ3	Rat	CTG​AAG​AAG​ACG​TTG​GGC​CT	TTG​GCC​CGT​CAA​TGT​CGT​AA
IL-6	Rat	CTT​CTT​GGG​ACT​GAT​GCT​GGT​GAC	AGG​TCT​GTT​GGG​AGT​GGT​ATC​CTC
IL-10	Rat	GGA​CAA​CAT​ACT​GCT​AAC​CGA​CTC	TGG​ATC​ATT​TCC​GAT​AAG​GCT​TGG

#### 2.6.2 Western blot analysis

Total protein was extracted from tissues or cultured cells and quantified using the bicinchoninic acid (BCA) assay (P0010S, Beyotime). Equal amounts of protein were separated via SDS–PAGE and transferred onto polyvinylidene fluoride (PVDF) membranes. After blocking with 5% non-fat milk, the membranes were incubated overnight at 4 °C with the following primary antibodies: THBS1 (1:1500, 18304-1-AP, Proteintech), PI3K (1:30000, 60225-1-Ig, Proteintech), p-PI3K (1:1000, CY6428, Abways), AKT (1:30000, 60203-2-Ig, Proteintech), p-AKT (1:5000, 66444-1-Ig, Proteintech), Collagen I (1:5000, 66761-1-Ig, Proteintech), Fibronectin (1:10000, 15613-1-AP, Proteintech), α-SMA (1:10000, 14395-1-AP, Proteintech), VEGF (1:2000, 66828-1-Ig, Proteintech), Integrin αvβ3 (1:2000, 18309-1-AP, Proteintech), IL-6 (1:1000, 30953-1-AP, Proteintech), and IL-10 (1:5000, 60269-1-Ig, Proteintech). The membranes were then incubated for 1 h at room temperature with horseradish peroxidase (HRP)-conjugated secondary antibodies (1:4000, YP848537, UpingBio; or 1:8000, BA1050, BOSTER). Protein bands were visualized using enhanced chemiluminescence (ECL).

### 2.7 Statistical analysis

Statistical analyses were performed using GraphPad Prism 10. Data are presented as the mean ± standard deviation (SD) derived from a minimum of three independent experiments. Group comparisons were conducted using one-way and two-way analysis of variance (ANOVA), with GAPDH serving as the internal control. A p-value of less than 0.05 was deemed statistically significant (*p < 0.05, **p < 0.01, ***p < 0.001, ****p < 0.0001).

## 3 Result

### 3.1 Transcriptome analysis

**FIGURE 2:**
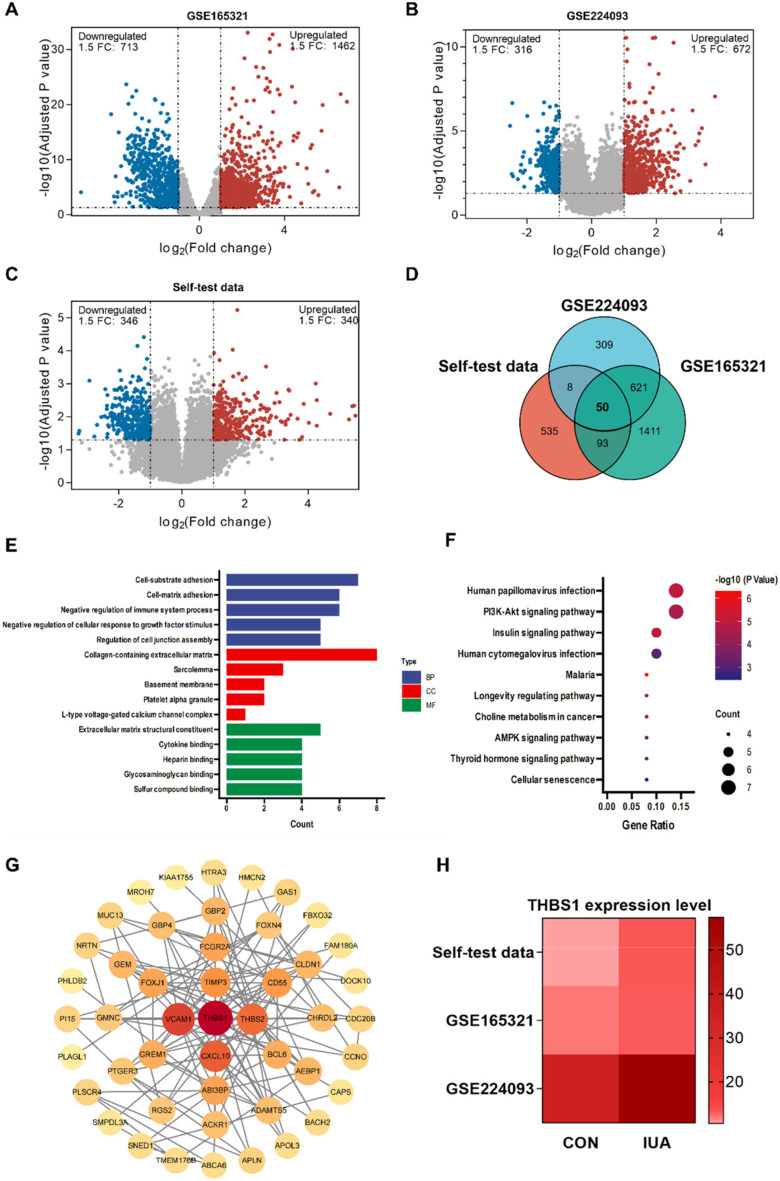
THBS1 as a key regulatory target in IUA progression. **(A–C)** Volcano plots showed differentially expressed genes (DEGs). **(D)** Venn diagram displaying the overlaps of 50 DEGs. **(E)** GO function analysis, including BP, CC, and MF. **(F)** KEGG pathway enrichment analysis of DEGs. **(G)** Protein-protein interaction (PPI) network. **(H)** Expression levels of THBS1 in self-test data, GSE165321, and GSE224093.

The study aimed to elucidate the molecular mechanisms underlying infertility caused by intrauterine adhesions (IUA). Endometrial tissues were collected from five IUA patients and five healthy controls for transcriptome sequencing. To validate the findings, the GEO datasets GSE224093 and GSE165321 were incorporated to increase the sample size. Differential expression analysis identified 50 differentially expressed genes associated with IUA (Figures 2A–D). GO function analysis revealed that these signature genes were significantly involved in the regulation of cell adhesion and extracellular matrix dynamics (Figure 2E). Meanwhile, KEGG pathway analysis indicated significant enrichment in the PI3K-AKT and AMPK signaling pathways, as well as metabolism-related pathways (Figure 2F). In-depth analysis revealed that the PI3K-AKT pathway might regulate cell-matrix interactions via integrin-mediated focal adhesion signaling. Additionally, its roles in angiogenesis and microenvironment homeostasis regulation were closely associated with molecular features such as glycosaminoglycan binding, highlighting its central role in the pathogenic mechanisms of IUA. Based on the PPI network analysis, we identified thrombospondin 1 (THBS1) as a key hub node within the network (Figure 2G). As a critical component of the extracellular matrix, THBS1 participates in tissue repair processes by mediating cell adhesion, vascular remodeling, and immune microenvironment regulation. Notably, significantly upregulated expression of THBS1 was observed in clinical samples and two independent datasets (Figure 2H), indicating its potential as a critical regulatory target in the progression of IUA. Collectively, these results suggest that THBS1 may be related to the development of IUA.

### 3.2 Establishment of the in vitro IUA cell model

**FIGURE 3:**
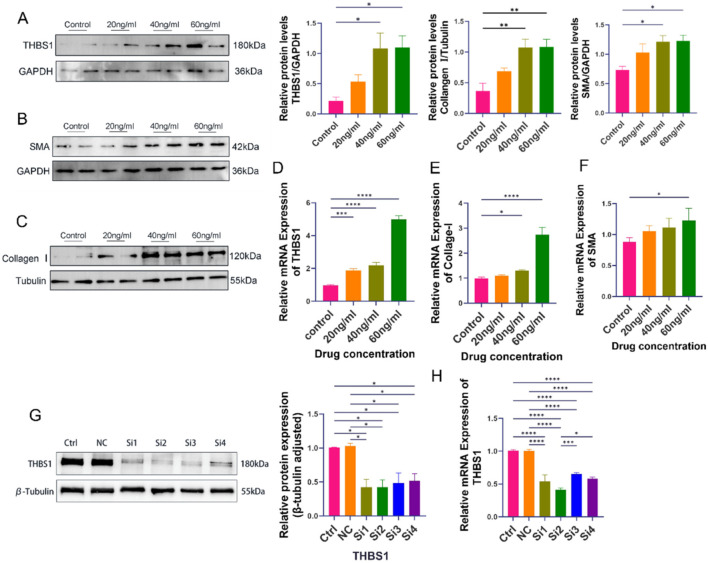
Establishment of the *in vitro* IUA cell model. IHESCs were stimulated with increasing concentrations of TGF-β1, and the expression levels of fibrosis-related markers were assessed. **(A)** Protein expression of THBS1. **(B)** Protein expression of α-SMA. **(C)** Protein expression of Collagen I. **(A–C)** The illustrated imprint graph originates from an independent test, representing two independent biological replicates. The quantitative data is based on a total of four independent biological replicates (i.e., two independent tests, each comprising two biological samples, n = 4). **(D–F)** mRNA expression of THBS1, Collagen I, and α-SMArespectively. In the control group, empty vector group, and siRNA-mediated THBS1 knockdown groups (Si1–4): **(G)** Protein expression of THBS1. **(H)** mRNA expression of THBS1. Data are presented as mean ± SD (n = 3). *P* < 0.05 (*), P < 0.01 (**), P < 0.001 (***)P < 0.0001 (****).

In the IHESC-based *in vitro* IUA model, both qRT-PCR and Western blot analyses revealed a dose-dependent increase in fibrosis severity with rising concentrations of TGF-β1 (Figures 3A –F). Notably, at a concentration of 60 ng/mL TGF-β1, the expression levels of fibrosis markers Collagen I and α-SMA, as well as the key pathogenic gene THBS1, were significantly elevated at both the mRNA and protein levels compared to the control group (Figures 3A–F). Based on these findings, 60 ng/mL TGF-β1 was selected for use in subsequent experiments. We employed siRNA to knock down the THBS1 gene in the IHESC cell line. Validation through qRT-PCR and WB revealed that si2RNA achieved the highest knockdown efficiency (Figures 3G,H). Consequently, we selected si2RNA for further experiments.

### 3.3 Roles of THBS1 and DMSCs in the in vitro IUA model

**FIGURE 4:**
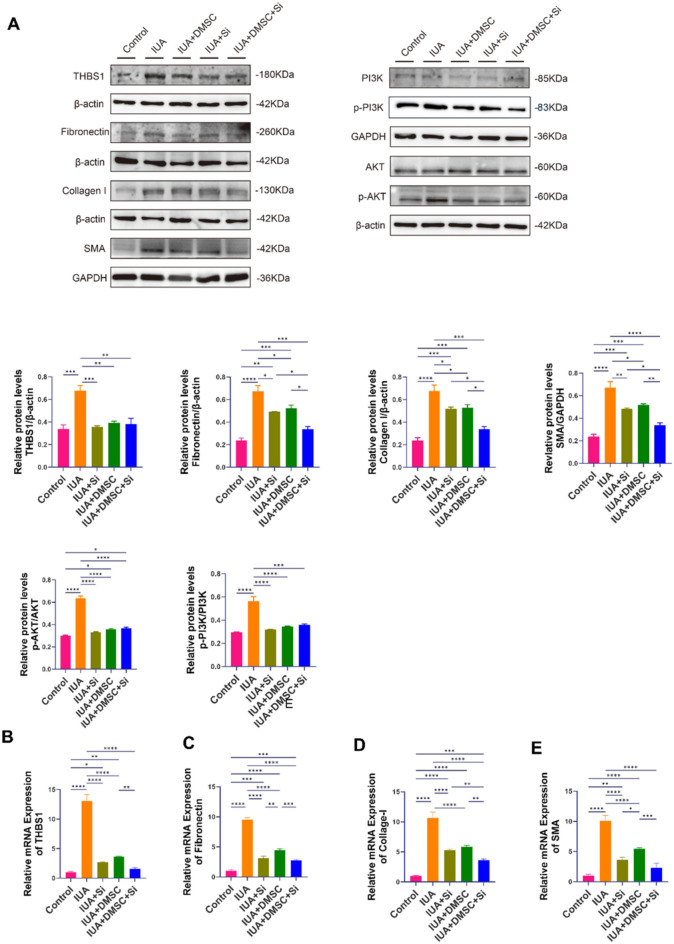
Effects of THBS1 and DMSCs in the *in vitro* IUA cell model. **(A)** Expression levels of fibrosis-related proteins and activation status of the PI3K/AKT signaling pathway across experimental groups. **(B–E)** mRNA expression levels of THBS1, Fibronectin, Collagen I and α-SMA, respectively. Data are presented as mean ± SD (n = 3). *P* < 0.05 (*), P < 0.01 (**), P < 0.001 (***)P < 0.0001 (****).

In the *in vitro* IUA cell model, both quantitative reverse transcription polymerase chain reaction (qRT–PCR) and Western blot analyses demonstrated a significant elevation in THBS1 expression in the IUA group compared to the control group (Figures 4A,B). Additionally, the expression levels of fibrosis-related markers, including Collagen I, α-smooth muscle actin (α-SMA), and Fibronectin, were significantly increased in the IUA group at both the mRNA and protein levels (Figures 4A,C–E). Concurrently, the phosphorylation levels of PI3K/AKT signaling proteins, specifically p-PI3K and p-AKT, were significantly upregulated (Figure 4A).

In both the THBS1 knockdown and DMSC-treated groups, the mRNA and protein expression levels of THBS1 expression was significantly reduced compared to the IUA group, which was accompanied by a downregulation of Collagen I, α-SMA, and Fibronectin (Figures 4A–E). Consistently, the phosphorylation levels of PI3K/AKT signaling proteins were also diminished (Figure 4B), further confirming that THBS1 regulates fibrosis progression through the activation of the PI3K/AKT pathway. Importantly, DMSCs appeared to attenuate fibrosis by suppressing THBS1 expression, thereby blocking downstream PI3K/AKT activation.

Notably, the expression of fibrosis-related genes in the DMSC-treated group was lower than that in the THBS1 knockdown group, suggesting that DMSCs exert anti-fibrotic effects through multiple regulatory mechanisms beyond mere THBS1 suppression.

In the combined DMSC and THBS1 knockdown group, the expression of THBS1 was comparable to that observed in the individual THBS1 knockdown and DMSC groups, aligning closely with baseline levels in the control group. However, the expression of fibrosis markers was significantly lower than in either single-treatment group (Figures 4A,B). These findings underscore the multifaceted anti-fibrotic properties of DMSCs and suggest that the combined approach of THBS1 knockdown and DMSC treatment provides synergistic efficacy in suppressing fibrosis in the IUA model.

### 3.4 Establishment of IUA and THBS1 knockdown animal models

#### 3.4.1 Establishment of the IUA rat model

**FIGURE 5:**
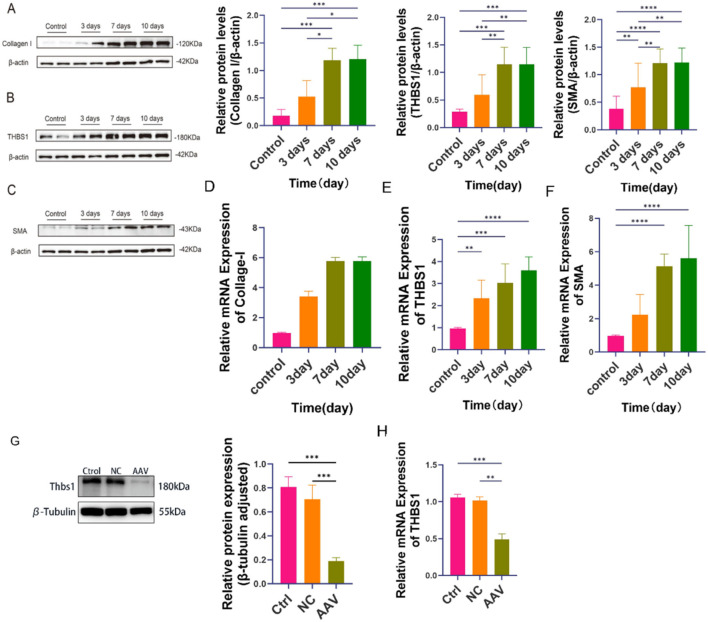
Establishment of the IUA and THBS1 knockdown animal models. **(A–C)** Protein expression levels of Collagen I, THBS1, and α-SMA in uterine tissues at 3, 7, and 10 days post-injury. The illustrated imprint graph originates from an independent test, representing two independent biological replicates. The quantitative data is based on a total of four independent biological replicates (i.e., two independent tests, each comprising two biological samples, n = 4). **(D–F)** Corresponding mRNA expression levels of Collagen I, THBS1, and α-SMA. **(G)** THBS1 Protein expression levels in the control, negative control (NC), and AAV-treated groups, confirming successful gene knockdown. **(H)** THBS1 mRNA expression levels in the control, negative control (NC), and AAV-treated groups, confirming successful gene knockdown.

In this study, we successfully established an intrauterine adhesion (IUA) animal model using a combined mechanical and chemical injury approach. We measured fibrosis-related gene and protein expression levels at 3, 7, and 10 days (n = 6) post-injury (Figures 5A–F). The results indicated a significant elevation in fibrosis on day 7, which appeared to stabilize at that time point. Consequently, subsequent therapeutic experiments were conducted using animals at day 7 post-injury as the standardized IUA model.

#### 3.4.2 Establishment of the THBS1 knockdown model

To downregulate THBS1 expression in the rat uterus, animals were randomly allocated into either a vehicle control group or an AAV group (n = 3 per group) and received a single intrauterine injection of adeno-associated virus (AAV) carrying shRNA sequences targeting THBS1. As depicted in Figures 5G,H, both THBS1 mRNA and protein levels in uterine tissues were markedly reduced after AAV administration, confirming the successful establishment of a THBS1-knockdown rat model.

### 3.5 Effects of THBS1 knockdown and DMSC treatment on the IUA rat model

**FIGURE 6:**
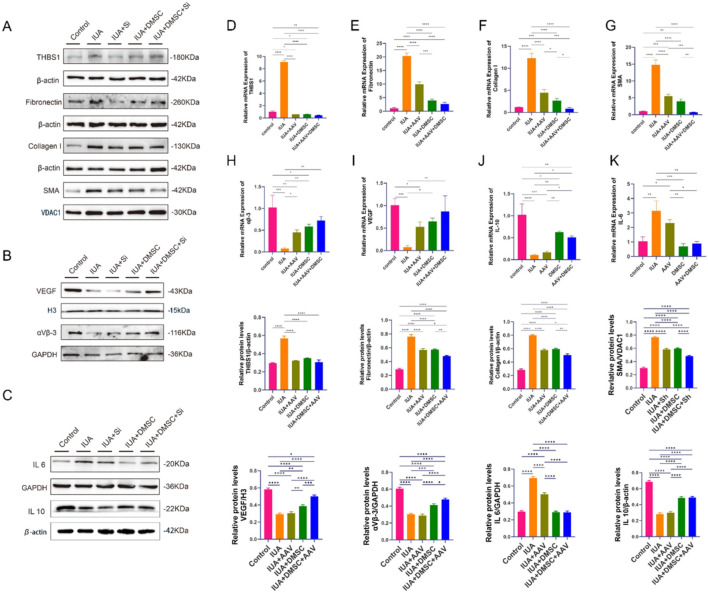
Effects of THBS1 knockdown and DMSC treatment on the IUA rat model. **(A)** Protein expression levels of fibrosis-related markers (Collagen I, α-SMA, and Fibronectin) across different treatment groups. **(B)** Protein expression levels of VEGF and integrin αvβ3. **(C)** Protein expression levels of inflammatory cytokines IL-6 and IL-10. **(D–K)** mRNA expression levels of THBS1 **(D)**, Fibronectin **(E)**, Collagen I **(F)**, α-SMA **(G)**, integrin αvβ3 **(H)**, VEGF **(I)**, IL-10 **(J)**, and IL-6 **(K)**. Data are presented as mean ± SD (n = 3). P < 0.05 (*), P < 0.01 (**), P < 0.001 (***)P < 0.0001 (****).

In the IUA animal model, both qRT-PCR and Western blot analyses demonstrated that THBS1 expression was significantly elevated in the IUA group compared to the control group (Figures 6A,B). The expression levels of fibrosis-associated markers—Collagen I, α-SMA, and Fibronectin—were also markedly increased in the IUA group. In all three treatment groups, THBS1 expression was significantly reduced relative to the IUA group, accompanied by a corresponding decrease in the expression of fibrosis markers (Figures 6A,B). These findings confirm the regulatory role of THBS1 in the progression of fibrosis.

Notably, elevated IL-10 expression was observed in both the DMSC-treated group and the combined treatment group, indicating the anti-inflammatory properties of DMSCs (Figure 6C). These results suggest that DMSCs may alleviate fibrosis not only by modulating the THBS1–PI3K/AKT signaling pathway but also by attenuating inflammatory responses. Regarding endometrial receptivity and angiogenesis, expression levels of VEGF and integrin αvβ3 were significantly higher in the DMSC and combined treatment groups than in the THBS1 knockdown group (Figure 6B). This highlights the role of DMSCs in promoting vascular remodeling and endometrial regeneration.

**FIGURE 7:**
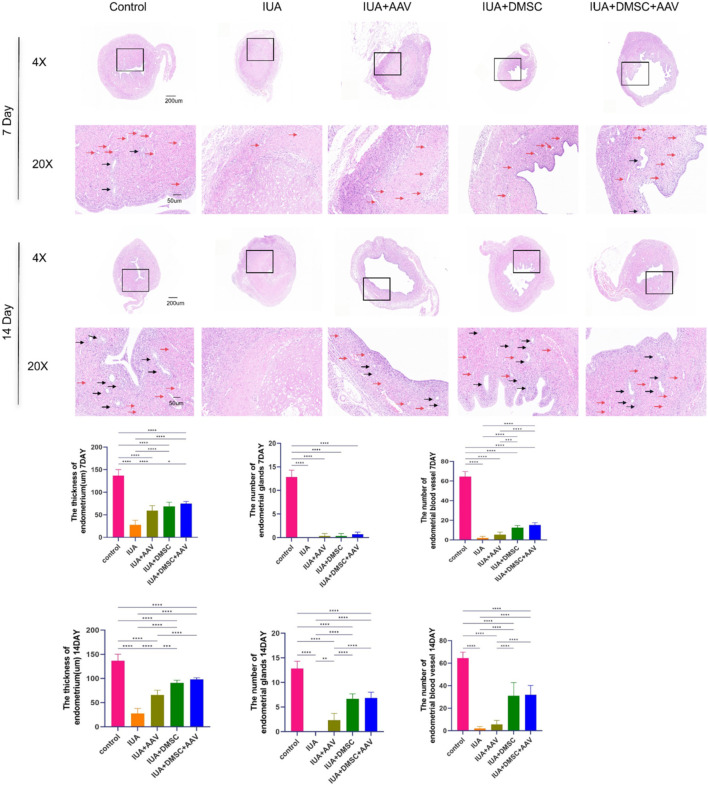
H&E staining of uterine tissues from each group. Representative images of H&E staining of uterine tissues from each group. Red arrows indicate blood vessels; black arrows indicate endometrial glands. *P* < 0.05 (*), P < 0.01 (**), P < 0.001 (***)P < 0.0001 (****).

**FIGURE 8:**
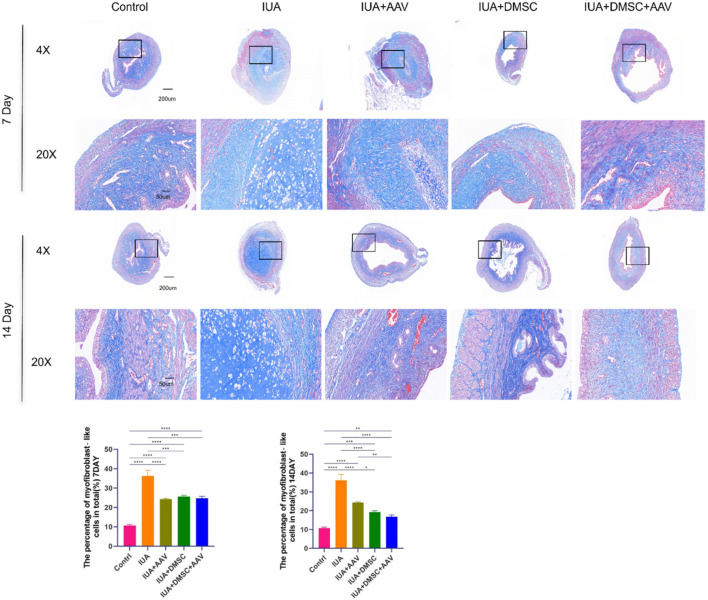
Masson staining of uterine tissues from each group. Representative images of Masson’s trichrome staining showing collagen deposition and fibrosis in the endometrium. Data are presented as mean ± SD (n = 6). *P* < 0.05 (*), P < 0.01 (**), P < 0.001 (***)P < 0.0001 (****).

To further assess the restoration of uterine cavity architecture, hematoxylin and eosin (H&E) staining, as well as Masson’s trichrome staining, were performed (Figures 7, 8). As described in previous studies (Thoprasert et al., 2023; Zhang et al., 2022; Zeng et al., 2025; Yanpeng et al., 2017), the thickness of the basal layer was measured on high-resolution hematoxylin and eosin (H&E) stained histological sections. Specifically, the measurement was taken from the basal epithelial-basal membrane junction to the deepest point of the basal layer. The measurements were conducted using ImageJ software. To ensure accuracy and consistency, at least three measurements were taken from non-overlapping fields within each section, and the average of these measurements was used to determine the final basal layer thickness. In the intrauterine adhesion (IUA) group, the uterine cavity was completely obliterated, characterized by a total loss of endometrial epithelial cells, significant thinning of the basal layer, and extensive accumulation of fibrotic connective tissue throughout the cavity (Figures 7, 8).

In the THBS1 knockdown group, no significant improvement was observed on day 7 post-treatment. By day 14, fibrosis was notably alleviated; however, the epithelial layer remained substantially deficient. This may be attributed to the relatively slow onset of adeno-associated virus (AAV)-mediated gene silencing and its limited capacity to promote endometrial regeneration (Figures 7, 8).

In contrast, both the DMSC-treated group and the combined treatment group exhibited significant reductions in fibrosis as early as day 7. Furthermore, partial restoration of the endometrial epithelium was observed, supporting the notion that DMSCs exert multifaceted therapeutic effects in IUA by inhibiting fibrosis and promoting tissue regeneration.

It is noteworthy that while the DMSC and combination therapy groups demonstrated substantial improvements compared to the IUA group, their outcomes remained statistically distinct from those of the uninjured control group. This discrepancy may be attributed to the limited retention time of transplanted DMSCs within the uterine environment. Future strategies that incorporate biomaterial-based scaffolds may enhance DMSC localization and survival, thereby improving therapeutic efficacy.

## 4 Disscussion

### 4.1 Mechanistic role of THBS1 in intrauterine adhesion (IUA)

In this study, transcriptomic analysis revealed a significant upregulation of THBS1 in the endometrial tissues of patients with IUA. Both *in vitro* and *in vivo* experiments confirmed that THBS1 plays a crucial role in promoting fibrosis by activating the PI3K/AKT signaling pathway. These findings are consistent with recent studies that highlight the fibrogenic function of THBS1 across various organ systems.

Hu et al. reported that THBS1 enhances TLR4-mediated M2 macrophage polarization and glycolytic activation, thereby facilitating the progression of pulmonary fibrosis (Hu et al., 2025). Similarly, Wei et al. demonstrated that silencing THBS1 suppresses Ang II-induced cardiac fibrosis in both cellular and animal models (Wei et al., 2024). Liu et al., utilizing spatial single-cell transcriptomics, showed that targeting THBS1 modulates M2 macrophage function and alleviates renal fibrosis (Liu J. et al., 2024). In the context of liver fibrosis, Cheng et al. found that THBS1 activates hepatic stellate cells and stimulates collagen production via the PI3K/AKT pathway (Cheng S. et al., 2023). Consistent with these findings, Kuer et al. observed that THBS1 knockdown reduced PI3K/AKT signaling, diminished fibrosis, and downregulated EMT-related proteins, ultimately attenuating renal fibrogenesis (Tuoheti et al., 2024).

These studies collectively suggest that THBS1 may promote fibrosis through its interaction with integrin receptors, which triggers downstream PI3K/AKT signaling and leads to the production and deposition of extracellular matrix (ECM).

Consistent with these findings, our results demonstrated that THBS1 knockdown *in vitro* significantly reduced the expression of fibrosis-related markers and inhibited PI3K/AKT activation. *In vivo*, THBS1 knockdown also mitigated endometrial fibrosis, reinforcing its pathological role in IUA. Taken together, our findings indicate that THBS1 promotes the activation of endometrial stromal cells and collagen synthesis via the activation of the PI3K/AKT pathway, thereby contributing to fibrosis and the development of intrauterine adhesions.

### 4.2 Therapeutic potential and mechanisms of MSC treatment

This study further investigates the therapeutic potential of mesenchymal stem cells (MSCs) in the context of intrauterine adhesion (IUA). Our findings indicate that MSC treatment significantly reduces THBS1 expression, inhibits the activation of the PI3K/AKT pathway, attenuates fibrosis, and enhances endometrial receptivity. These results align with previous research on the role of MSCs in fibrotic diseases. For instance, Cheng et al. demonstrated that MSCs mitigate liver fibrosis by releasing anti-fibrotic factors and inhibiting the YAP/LOXL2 signaling pathway (Cheng F. et al., 2023). Similarly, E et al. validated the anti-fibrotic effects of MSCs in models of renal fibrosis (Xiang et al., 2020). Furthermore, the anti-inflammatory and pro-angiogenic properties of MSCs have been extensively documented in the treatment of hepatic, pulmonary, cardiac, and uterine fibrosis (Zhang et al., 2023; Fang et al., 2025; Li et al., 2022; Liu B. et al., 2024; Harrell et al., 2024; Periera-Simon et al., 2021).

In line with these findings, our study demonstrated that MSC treatment not only suppressed THBS1 expression and PI3K/AKT activation but also significantly downregulated inflammation-related genes while upregulating VEGF expression. These results suggest that MSCs exert anti-fibrotic effects through multiple mechanisms: on one hand, they secrete soluble factors that inhibit THBS1 and PI3K/AKT signaling; on the other hand, they modulate the immune microenvironment through anti-inflammatory cytokines, thereby promoting endometrial repair. Additionally, MSC-derived VEGF may enhance angiogenesis, improve endometrial perfusion, and further support tissue regeneration.

### 4.3 Synergistic effects of THBS1 knockdown combined with MSC therapy

In our study, although the combined knockdown of THBS1 and MSC therapy did not further decrease THBS1 expression beyond that achieved by either treatment alone, the combination resulted in superior outcomes in fibrosis resolution and restoration of endometrial receptivity. This finding suggests a synergistic interaction between the two strategies. Specifically, THBS1 knockdown directly inhibits PI3K/AKT signaling, leading to reduced fibrosis, while MSCs provide additional therapeutic benefits through the secretion of anti-fibrotic and anti-inflammatory mediators, which further attenuate THBS1 signaling and enhance the regenerative microenvironment. Furthermore, the increased expression of VEGF likely facilitates neovascularization and improves endometrial blood supply, thereby promoting regeneration.

However, it is noteworthy that despite these improvements, the combined therapy group still exhibited statistically significant differences from the uninjured control group regarding endometrial thickness, gland density, and receptivity. This discrepancy may be attributed to limited MSC retention and suboptimal delivery efficiency *in vivo*. Future studies should explore the use of biomaterial scaffolds to enhance MSC localization, persistence, and therapeutic efficacy.

### 4.4 Clinical implications and future directions

This study identifies THBS1-mediated PI3K/AKT signaling as a central mechanism driving fibrosis in IUA and highlights THBS1 as a promising molecular target for therapeutic intervention. The data provide robust preclinical support for the development of combined THBS1-targeted and mesenchymal stem cell (MSC)-based strategies. These findings enhance our understanding of IUA pathogenesis and establish a theoretical foundation for novel treatment approaches.

Future research should focus on elucidating the precise molecular interactions between THBS1 and its upstream partners, such as integrin receptors, to clarify how these interactions regulate extracellular matrix synthesis and deposition. Additionally, optimizing MSC therapy—including dosing, timing, and delivery systems—will be crucial for improving therapeutic outcomes. Finally, well-designed clinical trials are necessary to assess the safety and efficacy of THBS1 knockdown in conjunction with MSC therapy in IUA patients, ultimately facilitating clinical translation.

## Data Availability

The original contributions presented in the study are included in the article/Supplementary Material. Further inquiries can be directed to the corresponding author.
